# Proteasomal Degradation of Proinsulin Requires Derlin-2, HRD1 and p97

**DOI:** 10.1371/journal.pone.0128206

**Published:** 2015-06-24

**Authors:** Hanneke Hoelen, Arnaud Zaldumbide, Wouter F. van Leeuwen, Ellen C. W. Torfs, Marten A. Engelse, Chopie Hassan, Robert Jan Lebbink, Eelco J. de Koning, Maaike E. Resssing, Arnoud H. de Ru, Peter A. van Veelen, Rob C. Hoeben, Bart O. Roep, Emmanuel J. H. J. Wiertz

**Affiliations:** 1 Department of Medical Microbiology, University Medical Center Utrecht, Utrecht, The Netherlands; 2 Department of Molecular Cell Biology, Leiden University Medical Center, Leiden, The Netherlands; 3 Department of Nephrology, Leiden University Medical Center, Leiden, The Netherlands; 4 Department of Immunohematology & Blood Transfusion, Leiden University Medical Center, Leiden, The Netherlands; Harvard Medical School, UNITED STATES

## Abstract

Patients with type 1 diabetes (T1D) suffer from beta-cell destruction by CD8^+^ T-cells that have preproinsulin as an important target autoantigen. It is of great importance to understand the molecular mechanism underlying the processing of preproinsulin into these CD8^+^ T-cell epitopes. We therefore studied a pathway that may contribute to the production of these antigenic peptides: degradation of proinsulin via ER associated protein degradation (ERAD). Analysis of the MHC class I peptide ligandome confirmed the presentation of the most relevant MHC class I-restricted diabetogenic epitopes in our cells: the signal peptide-derived sequence A15-A25 and the insulin B-chain epitopes H29-A38 and H34-V42. We demonstrate that specific silencing of Derlin-2, p97 and HRD1 by shRNAs increases steady state levels of proinsulin. This indicates that these ERAD constituents are critically involved in proinsulin degradation and may therefore also play a role in subsequent antigen generation. These ERAD proteins therefore represent interesting targets for novel therapies aiming at the reduction and possibly also prevention of beta-cell directed auto-immune reactions in T1D.

## Introduction

In type 1 diabetes patients (T1D), pancreatic beta cells are destroyed by autoreactive CD8^+^ T-cells that have preproinsulin as their most important target antigen [1]. The importance of these T-cells is emphasized by their presence in insulitic lesions and in peripheral blood of T1D patients [2, 3]. In mouse models, preproinsulin-derived peptides can be used to induce diabetes [4], whereas blocking immune responses to preproinsulin can prevent diabetes [1]. CD8^+^ T-cells were found to recognize several different sequences within the preproinsulin protein. Some CD8^+^ T-cell antigens originate from the signal sequence of preproinsulin [5], but the majority of the epitopes identified originate from the proinsulin protein itself [1]. In view of the dominant role of proinsulin as an autoantigen, it is of great importance to understand proinsulin degradation and its subsequent processing into peptides that are recognized by CD8^+^ T-cells.

**Fig 1:**
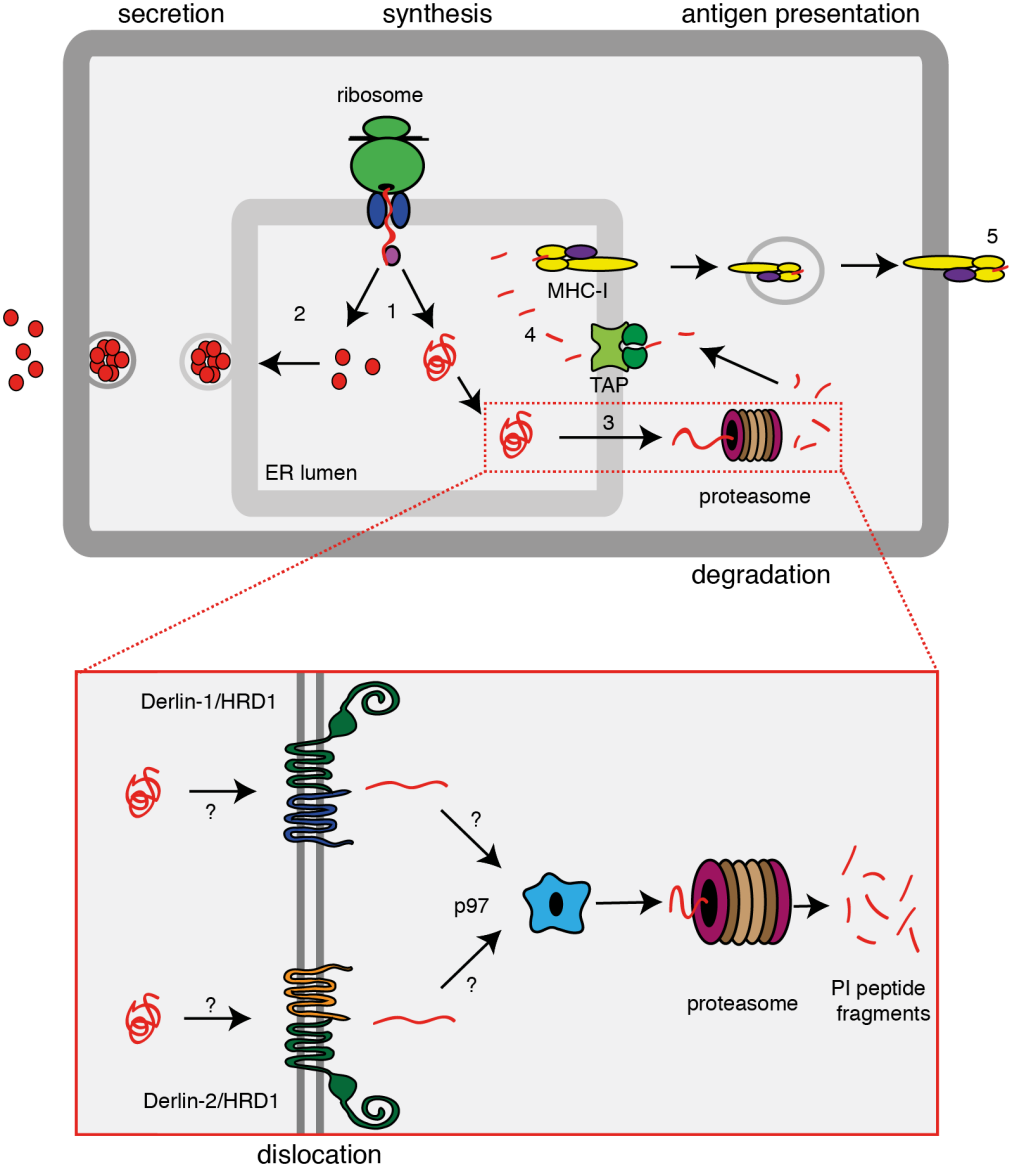
Insulin biosynthesis. Schematic representation of proinsulin synthesis, followed by either secretion or degradation. (1) Proinsulin molecules co-translationally translocate into the ER lumen. (2) Properly folded proinsulin molecules traffic to the secretory granules. Misfolded or abundant proinsulin molecules dislocate across the ER membrane (3) into the cytosol, potentially assisted by HRD1, Derlin-1 or Derlin-2 and p97 (inset). Cytosolic proinsulin is then degraded into smaller peptides by the proteasome. Proinsulin derived peptides are reimported into the ER lumen via TAP and (4) loaded onto MHC class I molecules. These traffic to the plasma membrane and (5) present the proinsulin-derived peptides to CD8^+^ T-cells.

The hormone precursor preproinsulin is co-translationally translocated into the ER lumen. After signal sequence cleavage and the formation of three disulfide bonds, the majority of the proinsulin molecules exit the ER and traffic via the Golgi to secretory granules. Within these granules, proinsulin is cleaved into the insulin A-B chain dimer and C-peptide. In response to blood glucose levels, insulin is secreted into the extracellular environment (Fig 1, left part). In addition to exit from the ER via the secretory pathway, proinsulin may enter the ER associated protein degradation (ERAD) pathway (Fig 1, right part). It has been estimated that 30–50% of all newly synthesized proteins are degraded immediately after their completion [6]. The proportion of newly synthesized proinsulin that is degraded in pancreatic β-cells is unknown but, considering the large quantities of insulin these cells secrete [7], it is very likely that significant amounts of proinsulin are degraded.

Degradation of ER luminal and membrane proteins occurs via the ER Associated protein Degradation (ERAD) pathway [8]. ERAD-clients are unfolded and reduced by ER-resident chaperones and oxidoreductases and subsequently dislocated (retro-translocated) across the ER membrane into the cytosol, where they are degraded by the proteasome. On their way to the proteasome, proteins are ubiquitinated by E3 ligases. Several ER-membrane E3 ligases have been identified, of which gp78 [9], HRD1 [10], TEB4 [11], TRC8 [12] and TMEM129 [13, 14] are the most characterized. HRD1 has been implicated in the degradation of mouse proinsulin [15]. Its yeast homologue, Hrd1p, has been suggested to form the pore through which ER luminal ERAD substrates dislocate [16, 17]. HRD1 forms complexes with the membrane proteins Derlin-1 and Derlin-2 [18, 19] (Fig 1 inset). Although the specific role of Derlin-1 and Derlin-2 in the ERAD pathway still remains to be determined, these proteins have been found to be essential for dislocation of several ERAD-clients into the cytosol [20, 21]. At present, it is unknown if any of these Derlin proteins are required for the dislocation and/or degradation of proinsulin.

For a number of degradation substrates, extraction from the ER membrane has been shown to require the AAA-ATPase p97 (VCP) [22]. P97 shuttles substrates from the membrane to the proteasome for degradation into smaller peptides. The resulting peptides may be reimported into the ER lumen by the TAP transporter and may subsequently be loaded onto MHC class I molecules for presentation to CD8^+^ T-cells (Fig 1, right part). In the view of the important role of CD8^+^ T-cells in the etiology of T1D it is important to understand the molecular mechanism of insulin degradation, including the role of ERAD in this process.

We recapitulate the ER stages of proinsulin biogenesis using a surrogate beta-cell as a study model. Elution of peptides from MHC class I molecules isolated from these cells confirms the presentation of the most relevant MHC class I diabetogenic epitopes: the signal peptide-derived sequence A15-A25 and the insulin B-chain epitopes H29-A38 and H34-V42. We demonstrate in these cells that specific silencing of Derlin-2, p97 and HRD1 by shRNA increases steady state levels of proinsulin, indicating that these ERAD proteins are involved in proinsulin degradation and subsequent antigen generation.

## Results

### Proinsulin degradation products are loaded onto MHC class I molecules

**Fig 2:**
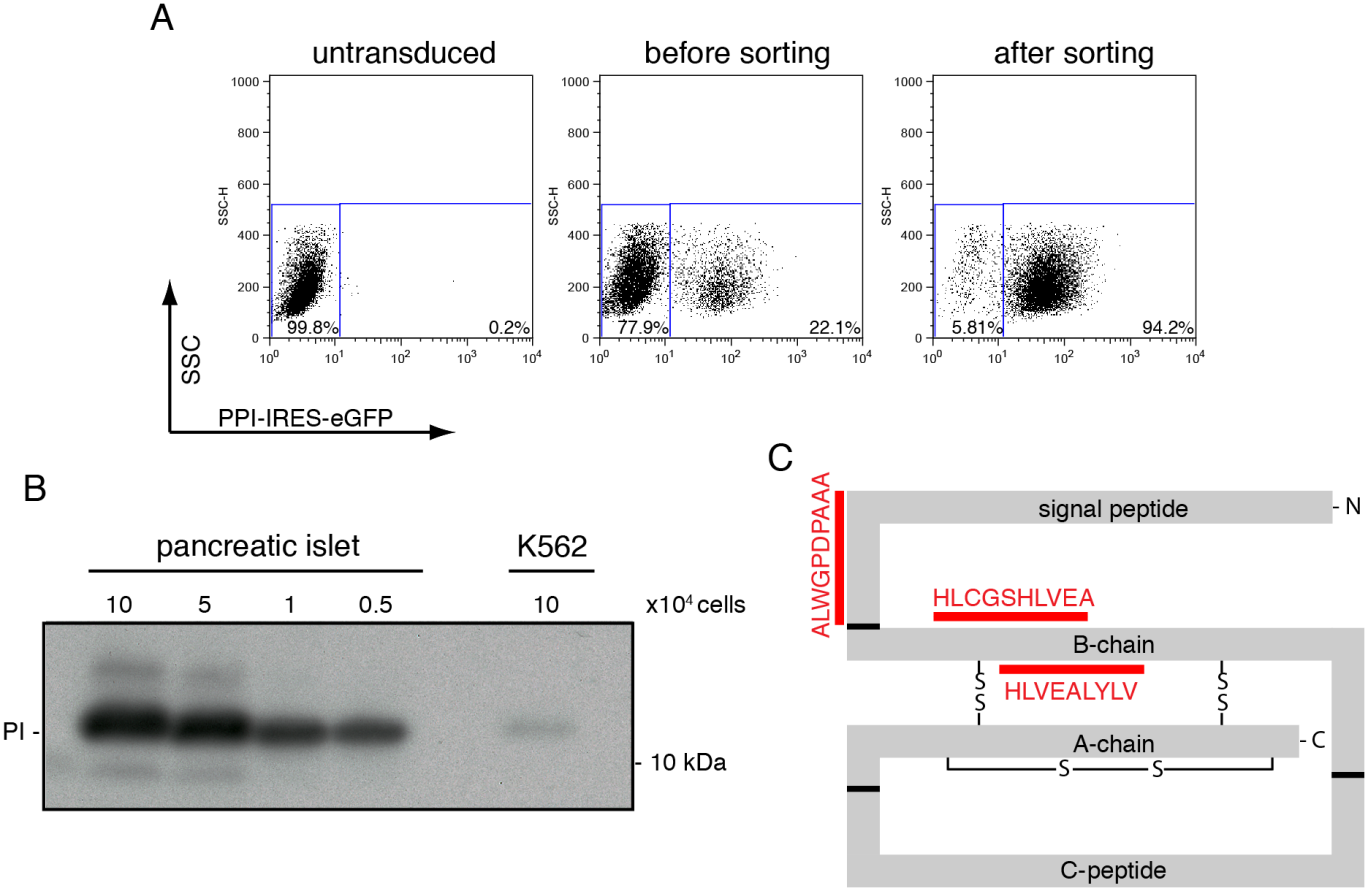
Generation of preproinsulin expressing K562 cells. (A) K562 cells were retrovirally transduced to express preproinsulin-IRES-GFP and sorted for the GFP positive population. Flow cytometry analysis of wild-type preproinsulin-expressing cells before transduction (left panel), after retroviral transduction but before sorting (middle panel) and after sorting (right panel). Sorting yielded a cell population that was approximately 95% GFP positive. (B) Human pancreatic islets cells and preproinsulin-expressing K562 cells were lysed and proteins were separated on 12% Nu-PAGE. Proinsulin levels were analyzed by Western blot. The number of cells used to prepare the lysates is indicated. (C) Schematic representation of the preproinsulin molecule including the three disulfide bonds. The epitopes eluted from MHC class I molecules are depicted in red.

To study the processing of proinsulin into antigenic peptides, a myelogenous leukemia cell line, K562, that stably expresses MHC class I (HLA-A2) [23], was transduced with retroviruses carrying the (mutant) preproinsulin gene followed by an internal ribosome entry site (IRES) and an eGFP-encoding sequence. This resulted in a pool of eGFP-positive cells that, after FACS sorting, were 95% positive for eGFP (Fig 2A). To evaluate the proinsulin levels in the K562 cells compared to pancreatic β-cells, lysates from preproinsulin-expressing K562 cells and from human islets were loaded onto SDS-PAGE and immunoblotted for proinsulin (Fig 2B). While proinsulin was abundantly present in the human islet cells, only a small quantity was detectable in the K562 cells, indicating that proinsulin is expressed in the surrogate β-cells, but at levels that are much lower compared to those in human islet cells.

Presentation of proinsulin-derived peptides to T-cells requires loading of the peptides onto MHC class I molecules (Fig 1, right part). To investigate whether peptides resulting from proinsulin degradation also follow this pathway in our K562 cells, we affinity purified MHC class I molecules from GFP-sorted cells. The peptide ligandome was acid-eluted from the MHC class I molecules and analyzed by mass spectrometry. This revealed three peptides that were derived from preproinsulin (Table 1 and Fig 2C and S1 Fig). One peptide originated from the signal sequence (A15-A24); this peptide has been identified previously as an important autoantigen in T1D [5]. CD8^+^ T-cells recognizing this epitope destroy β-cells in a glucose-dependent fashion. The second and third peptide, H26-A38 and H34-V42, both originate from the B-chain of proinsulin. The H34-V42 peptide has been identified as a CD8^+^ T-cell epitope in diabetes-specific immune responses [24, 25]. These findings show that the preproinsulin-expressing K562 cells produce biologically relevant epitopes recognized by diabetic CD8^+^ T-cells, which indicates that these cells may serve as a suitable model system to study the generation of these epitopes.

**Table 1:** Epitopes eluted from MHC class I.

Epitope	Sequence
PPI^15–24^ (SP15-24)	ALWGPDAAA
PPI^29–38^ (B5-14)	HLCGSHLVEA
PPI^34–42^ (B10-B18)	HLVEALYLV

### Proinsulin is degraded by the cytosolic proteasome

**Fig 3:**
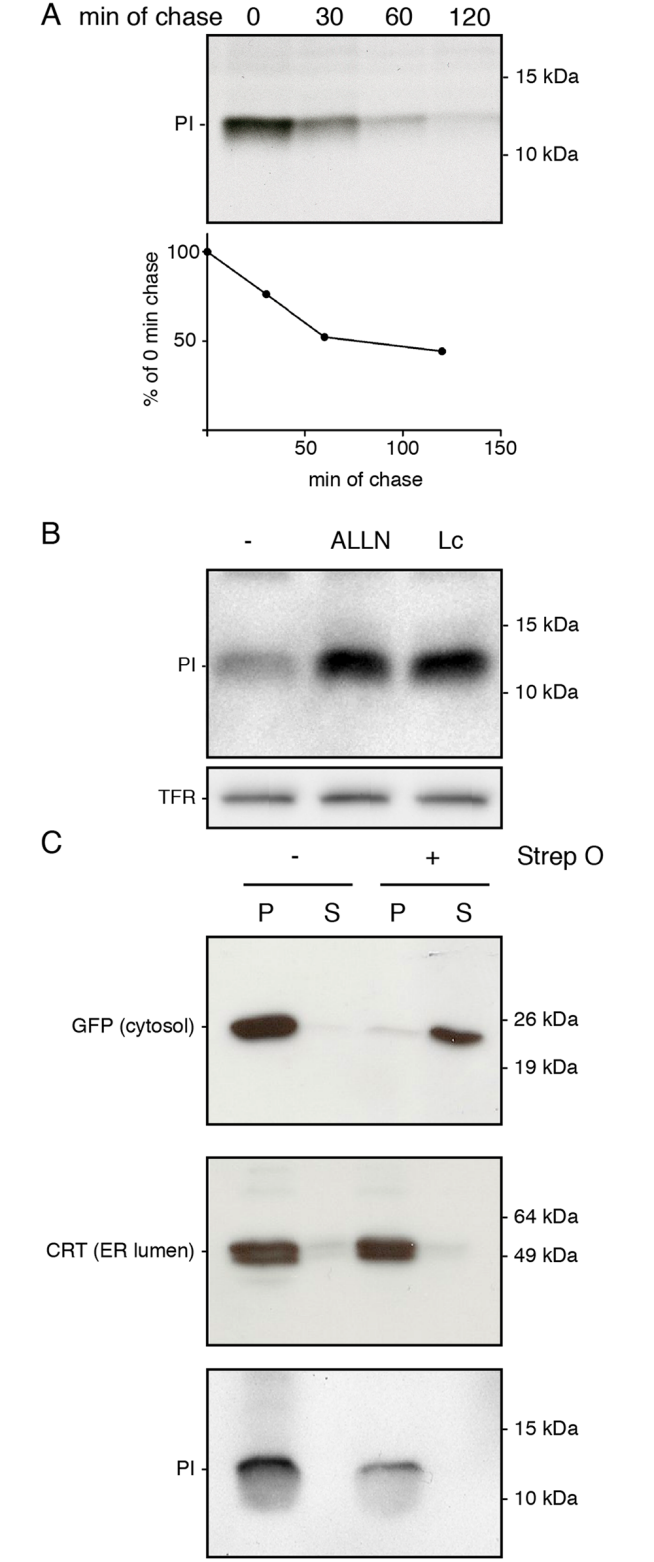
Proinsulin is dislocated into the cytosol and degraded by the proteasome. (A) K562 cells stably expressing preproinsulin were pulse labeled with ^35^S-methionine and cysteine for 15 minutes and chased for the indicated times. Immunoprecipitated proinsulin was analyzed using 15% SDS-PAGE. Quantification of the pulse chase experiment is shown below. Gel is representative for four experiments. (B) Preproinsulin-expressing K562 cells were treated for 3 hours with either DMSO, 100 μM ALLN or 10 μM Lactacystin (Lc). Cells were lysed and proteins were separated using 12% Nu-PAGE. Proinsulin levels were analyzed by Western blot. Transferrin receptor (TFR) was blotted as a loading control. Gel is representative for four experiments. (C) K562 cells stably expressing preproinsulin were treated for 3 hours with 100 μM ALLN and treated with Streptolysin-O to permeabilize the plasma membrane. After separation of the cytosol (supernatant) from the cell (pellet), protein levels for GFP (top panel), calreticulin (CRT, middle panel) and proinsulin (PI) were analyzed using 15% SDS-PAGE and Western blot. Gels are representative for three experiments.

Pulse chase assays were used to study proinsulin maturation. K562 cells were pulse-labeled for 15 minutes with ^35^S-labeled methionine and cysteine and chased for the times indicated (Fig 3A). Analysis of immunoprecipitated proinsulin via SDS-PAGE revealed that the radiolabeled preproinsulin molecules largely disappeared within two hours after synthesis. In pancreatic β-cells, convertases catalyze the excision of the C-peptide from proinsulin resulting in the A-B chain heterodimer [26]. Since these convertases are absent in K562 cells, proinsulin remains unprocessed in these cells. The disappearance of proinsulin in the K562 cells can therefore either be explained by secretion or by degradation. No radiolabeled proinsulin was detected by immunoprecipitation from the supernatant after the two hours of chase (data not shown). Therefore, its unlikely that secretion accounts for the complete loss of proinsulin during the two hours chase period. Since we did not observed cell-death during the experiment either, we hypothesized that the proinsulin molecules disappeared due to degradation.

To assess whether proinsulin is degraded in a proteasome-dependent manner, K562 cells were treated with two compounds that inhibit the proteasome, N-Acetyl-Leu-Leu-Norleu-al (ALLN) and Lactacystin (Lc). This resulted in a significant increase of proinsulin compared to the DMSO (-) control (Fig 3B). Because the K562 cells showed no detectable secretion of proinsulin and displayed high levels of proteasome dependent degradation, these cells were used as model system to study the proinsulin degradation pathway in more detail.

Because the signal sequence of preproinsulin targets the protein into the ER lumen co-translationally and since the proteasome resides in the cytosol, degradation of proinsulin would require its dislocation across the ER membrane. To monitor dislocation of proinsulin, K562 cells were treated with proteasome inhibitors after which the plasma membrane was permeabilized with Streptolysin O. The supernatant (soluble cytosolic fraction) was separated from the pellet (ER lumen and membrane fractions) through centrifugation and protein content was analyzed by Western blotting (Fig 3C). The cytosolic GFP protein appeared only in the supernatant fraction whereas calreticulin (CRT), a soluble ER luminal protein, was solely visible in the pellet fraction, indicating that only the plasma membrane was permeabilized while the ER membrane remained intact. Proinsulin was exclusively detected in the pellet fraction. Because the proinsulin that accumulates after proteasome inhibition remains in a membrane-enclosed compartment (Fig 3C) and since we could not detect any secretion of proinsulin in our pulse chase assays (Fig 3A), the proinsulin most likely remained within the ER lumen to await eventual dislocation into the cytosol and degradation by the proteasome. The finding that proteasome inhibition results in a block in dislocation is not unique and has been observed for other ERAD-substrates previously [27]. The fact that proinsulin was found in the ER luminal fraction confirms that the proinsulin molecules enter the ER lumen initially and require dislocation out of the ER lumen for degradation via the ERAD pathway. The observed ER-luminal accumulation of proinsulin upon proteasomal inhibition suggest tight coupling of the ubiquitin-proteasome system to the dislocation pathway.

### Proinsulin degradation depends on Derlin-2, HRD1 and p97

**Fig 4:**
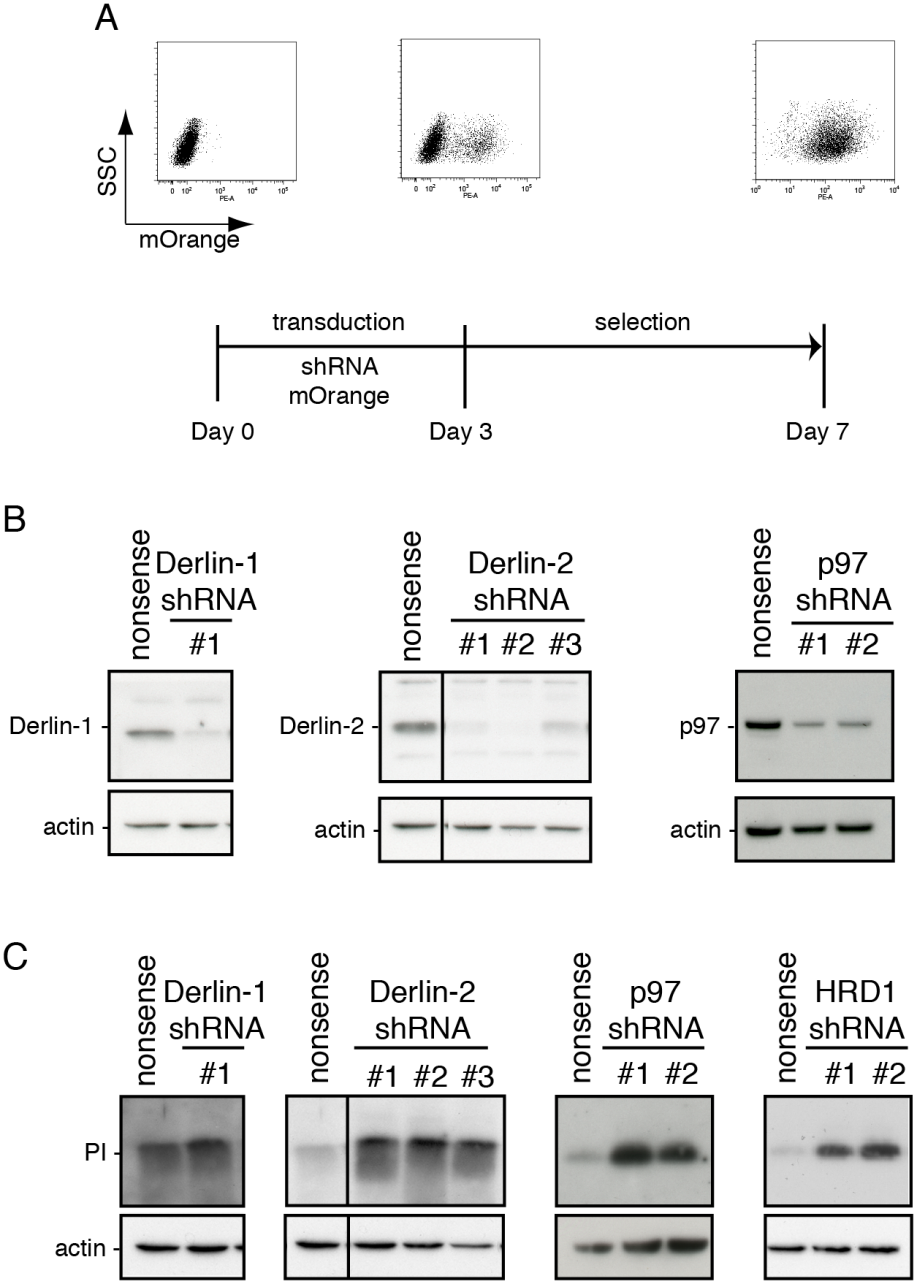
Derlin2, p97 and HRD1 knockdown increases proinsulin steady state levels. (A) Schematic representation of the experimental setup for the knockdown of ERAD proteins with shRNAs. Preproinsulin-expressing K562 cells were transduced to express the respective shRNAs together with mOrange from a bicistronic lentiviral expression vector. mOrange expression levels were analyzed by flow cytometry either before transduction or on day 3 and day 7 after transduction; the latter includes 4 days of selection with puromycin. Flow cytrometry analysis of a representative transduction of K562 cells is shown. (B) Proinsulin-expressing K562 cells were transduced with the indicated shRNAs. Seven days after transduction, cell lysates were prepared and loaded onto 12% Nu-PAGE; Derlin-1, Derlin-2 and p97 protein levels were analyzed by Western blot. Actin was included as a loading control. Gels are representative for three different experiments. (C) K562 cells were transduced as described for B and proinsulin levels were analyzed by Western blot. Actin was included as a loading control. Gels are representative for three different experiments.

Proteasomal degradation of proteins residing in the ER lumen occurs via the ERAD pathway. To determine which proteins of the ERAD pathway are involved in proinsulin dislocation, we generated a set of lentiviral vectors containing shRNAs directed against Derlin-1, Derlin-2, p97 and the ER membrane-localized E3 ligase HRD1 (Fig 1, inset). The viral vectors co-expressed a puromycin selection gene and the mOrange protein to monitor transduction efficiency. After seven days of selection, flow cytometry analysis showed that 95–100% of the cells were mOrange-positive (Fig 4A). The shRNAs against Derlin-1, Derlin-2 and p97 caused a significant decrease of their respective target proteins compared to a random control shRNA (Fig 4B). Verification of HRD1 knockdown at the protein level was not possible because intracellular HRD1 levels were too low to be detected by Western blot analysis (data not shown).

After verifying knockdown of the ERAD components, the same cell lysates were blotted for proinsulin (Fig 4C). Derlin-1 knockdown had no clear effect on proinsulin levels. In contrast, Derlin-2, p97 and HRD1 knockdown resulted in a strong increase of proinsulin levels. These findings indicate that Derlin-2, HRD1 and p97 are involved in the degradation of proinsulin.

### Increased expression of Derlin-2 reduces steady-state levels of proinsulin

**Fig 5:**
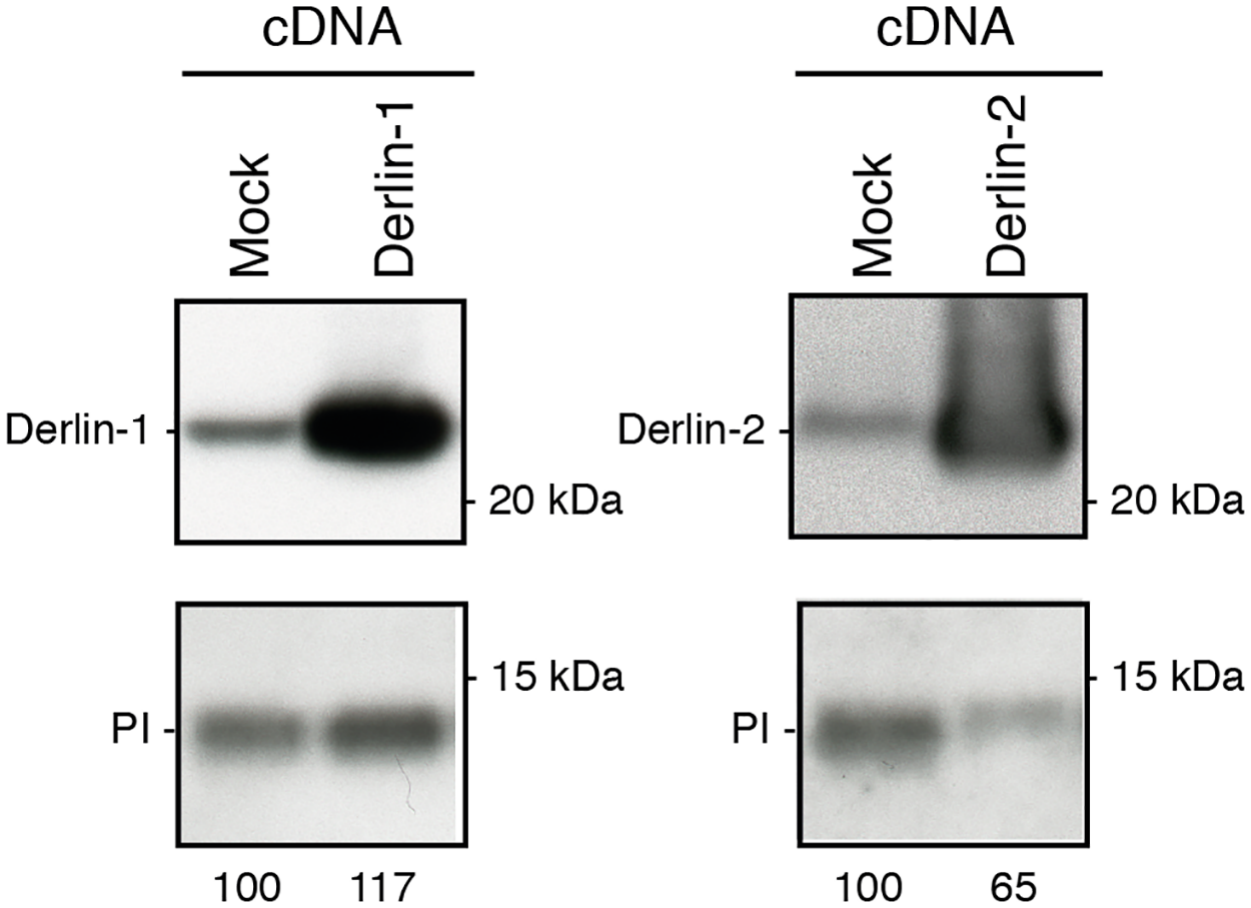
Derlin-2 overexpression decreases proinsulin steady state levels. K562 cells stably expressing preproinsulin were transduced with cDNA to overexpress Derlin-1 or Derlin-2 from a lentiviral expression vector. Seven days post transduction and selection, cell lysates were prepared and separated on 12% Nu-PAGE. Protein levels were analyzed by Western blot using antibodies against the indicated proteins and quantification of PI levels is shown. Gels are representative for three different experiments.

Because lowering protein levels of Derlin-2 resulted in increased proinsulin levels, we anticipated that overexpressing this ERAD component might create an opposite effect and might promote proinsulin degradation. To test this, we overexpressed Derlin-1 and Derlin-2 in preproinsulin-expressing K562 cells and subsequently monitored the relative levels of proinsulin in these cells via Western blotting (Fig 5). Derlin-1 and Derlin-2 both showed a strong increase in protein levels upon overexpression (Fig 5 upper panels) although lanes were loaded with an equal amount of cellular protein (data not shown). Increase of Derlin-1 protein expression slightly increased proinsulin levels,. Yet, overexpression of Derlin-2 resulted in decreased proinsulin levels (Fig 5, lower panels). In summary, knockdown and overexpression of Derlin-2 increase and decrease proinsulin protein levels, respectively, indicating that Derlin-2 has a strong effect on the level of proinsulin degradation in K562 cells.

### Derlin-2 depletion reduces proinsulin degradation

**Fig 6:**
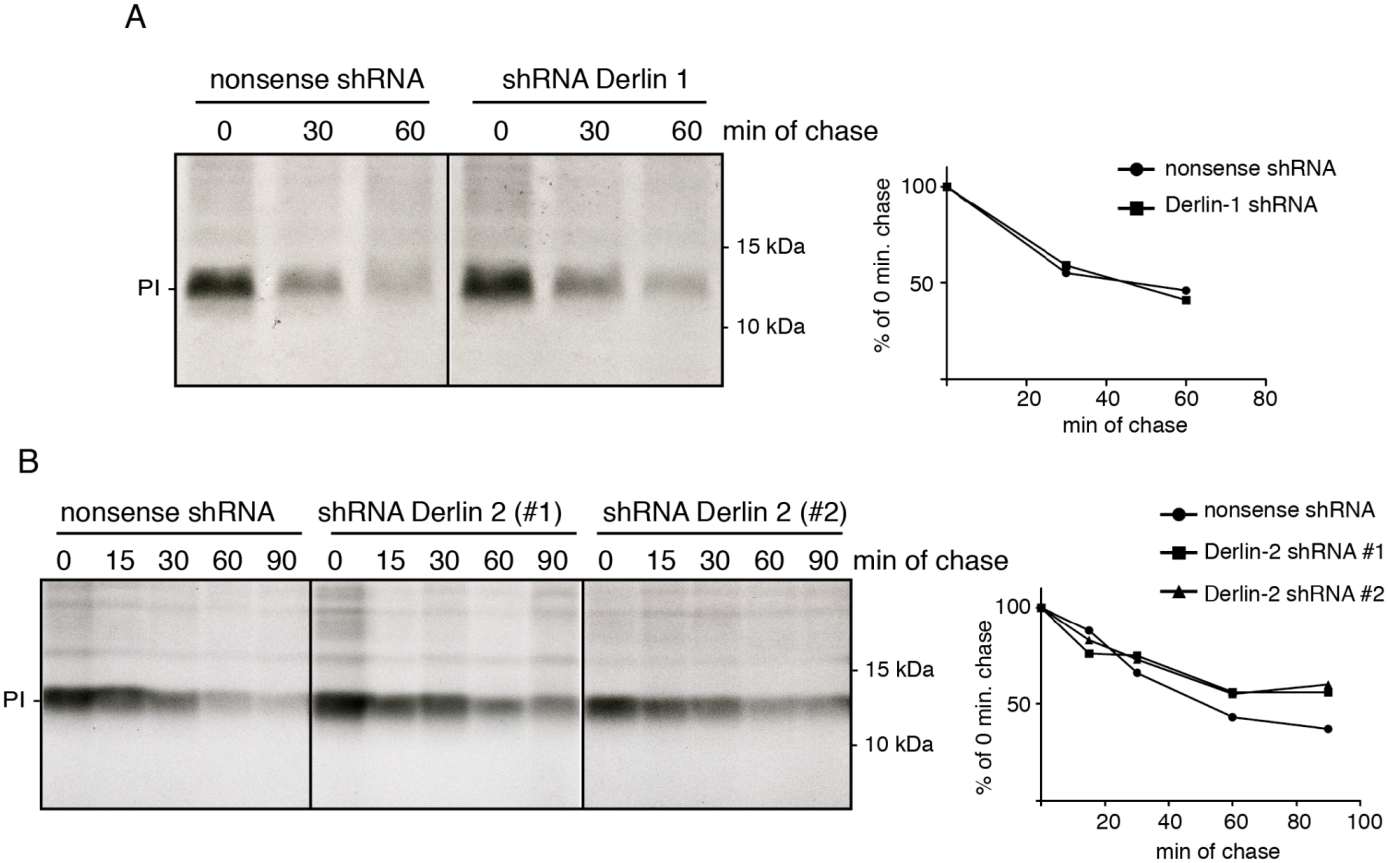
Derlin-2 depletion delays insulin degradation. (A) K562 cells stably expressing preproinsulin were transduced to express either nonsense shRNA (left) or an shRNA targeting Derlin-1 (right). After pulse-labeling for 15 minutes with ^35^S-methionine and cysteine, cells were chased for the indicated times. Proinsulin was immunoprecipitated from the lysates and analyzed using 15% SDS-PAGE. Quantification of the pulse chase experiment is shown on the right. Gels are representative for two different experiments. (B) Similar as described for A but with two shRNAs targeting Derlin-2. Quantification of the pulse chase experiment is shown on the right.

Because Western blots only reflect steady-state conditions, proinsulin degradation kinetics were studied in a pulse chase assay. Seven days post transduction of Derlin-1 and Derlin-2 shRNAs, preproinsulin-expressing K562 cells were pulse-labeled and proinsulin degradation was monitored in time. None of the shRNAs had a significant effect on the de novo synthesis as indicated by the comparable proinsulin levels in the different cells directly after the pulse (Fig 6). Knockdown of Derlin-1 had no detectable effect on proinsulin degradation (Fig 6A). Knockdown of Derlin-2 using two different shRNAs resulted in a significant increase in proinsulin levels, especially after 60 and 90 minutes of chase (Fig 6B). These findings indicate that proinsulin degradation is inhibited in K562 cells upon silencing of the Derlin-2 gene using shRNAs.

## Discussion

In view of the emerging role of proinsulin as an autoantigen in T1D, this study focuses on the processing of proinsulin into epitopes for recognition by CD8^+^ cytotoxic T-cells. Proinsulin is processed into three clinically relevant T-cell epitopes in our surrogate cells we using shRNA gene silencing we identified Derlin-2, HRD1 and p97 to be involved in proinsulin degradation.

Eluting the MHC class I ligandome and subsequent mass spectrometry analysis revealed that preproinsulin was processed into at least three different CD8^+^ T-cell epitopes in our surrogate beta-cells. These three specific peptide sequences are clinically relevant and their corresponding CD8^+^ T-cells are found in T1D specific immune responses [5, 24, 25]. Furthermore, the B10-B18 (H34-V42) epitope that we found is, albeit shorter, homologues to the mouse B9-B23 epitope that is accountable for the diabetic phenotype of the non-obese diabetic (NOD) mouse [28–30]. The list of identified preproinsulin-derived CD8^+^ T-cell epitopes that give rise to a diabetes-specific immune response is dominated by sequences originating from the B-chain of proinsulin [1]. Given that the B-chain epitopes are produced via proteasomal degradation (Fig 1) is it not surprising that the B-chain of proinsulin harbors the majority of the proteasomal cleavage sites predicted within the proinsulin molecule [25, 31].

Inhibition of the proteasome resulted in an increase of steady-state proinsulin levels in our cells. The proteasomal degradation of proinsulin is not restricted to K562 cells and is in line with previous observations in 293T cells [32], COS7 cells [15] and rat pancreatic islets [33]. Our study indicates that inhibition of the proteasome results in a block of proinsulin dislocation into the cytosol. This causes an accumulation of proinsulin within a membrane-enclosed cellular compartment, presumably the ER lumen. This tight coupling between dislocation and degradation is also observed for MHC class I molecules after β2m depletion and proteasome inhibition [27]. Although the reason for this tight coupling between dislocation and degradation is unknown, it may represent a mechanism to prevent accumulation of undigested proteasome substrates in the cytosol, where they potentially may form toxic aggregates [34].

Using shRNA gene silencing we found that downregulating Derlin-2, HRD1 and p97 increased steady-state levels of proinsulin, indicating that these proteins facilitate proinsulin degradation. Knockout of the Derlin-1 and Derlin-2 genes causes embryonic lethality in mice [35, 36], stressing their importance for cellular functioning. Despite this importance, only a small pool of mammalian ERAD substrates are identified that rely specifically on one the Derlins for their degradation, suggesting reduncency between these homologues. Proinsulin and sonic hedgehog protein [37] are currently the only two known non-glycosylated ER luminal ERAD-substrates that rely solely on Derlin-2 for their degradation. The fact that both substrates are non-glycosylated advocates towards a more general role for Derlin-2 in ERAD, not restricting it to glycosylated clients as suggested previously [38].

Both Derlin-1 and Derlin-2 are found in a HRD1-containing complex, which also contains the ERAD constituents Sel1L, Herp, VIMP (VCP interacting membrane protein) and p97 [18, 37, 39]. The degradation of proinsulin was sensitive to the down-regulation of both Derlin-2 and HRD1 but not Derlin-1. Although both Derlins are present in this complex, the interaction reported for Derlin-2 with HRD1 appears stronger then the interaction of Derlin-1 with HRD1 [37]. Also, Derlin-2 seems the major Derlin constituent in the HRD1-complex [18]. These conditions may explain that HRD1 acts in concert with Derlin-2 to dislocate and degrade ERAD-clients rather then together with Derlin-1.

Derlin-2 and p97 have been identified in a complex with the soluble ER-luminal protein SDF2L1. SDF2L1 was also found to bind a folding-defective mutant form of proinsulin [40]. Intriguingly, knockdown of SDF2L1 has an opposite effect to what we found for Derlin-2, since it rather increased proinsulin degradation. The latter suggests a role for SDF2L1 in limiting the accessibility of proinsulin to ERAD for instance by retaining proinsulin within the ER lumen to favor its folding. This potential role for SDF2L1 as a chaperone for proinsulin fits with the observation that SDF2L1 is present in a chaperone-complex together with several other ER-resident chaperones [41].

The insulin producing beta-cells of T1D patients are reported to suffer from chronic ER stress [42]. Most ERAD proteins, including Derlin-2, HRD1, p97 and SDF2L1, are upregulated under ER stress conditions [10, 38, 43, 44]. This increase in ERAD-protein expression ensures that accumulating ERAD substrates are cleared from the ER lumen and are targeted for degradation. The K562 cells do not express preproinsulin endogenously and therefore probably will display altered degradation efficiency and kinetics. But because the ERAD pathway and expression of all its constituents is conserved throughout all cell types, we expect that the degradation route of proinsulin and its requirement of Derlin-2, p97 and HRD1 will be similar in beta-cells.

It has been widely accepted that degradation products of newly synthesized proteins are the source for epitopes presented to CD8^+^ T-cells in MHC class I molecules. Decreasing proinsulin degradation in pancreatic β-cells therefore potentially opens new avenues for treatment of type 1 diabetes, as blocking proinsulin degradation may affect the presentation of proinsulin-derived epitopes via MHC class I molecules to CD8^+^ T-cells [45]. Prevention of ERAD through small compounds like eeyarestatin may decrease proinsulin degradation [46]. The latter may also positively influence insulin secretion because more proinsulin molecules may enter the secretory route. The latter has been reported for rat β-cells where proteasome inhibition with lactacystin resulted in increased insulin secretion under high glucose conditions [33].

Our study revealed that Derlin-2, p97 and HRD1 play an important role in proinsulin degradation. Inhibition of these ERAD constituents may reduce the generation of insulin-derived auto-antigens. The same time, secretion of insulin may be increased. Thus Derlin-2, HRD1 and p97 represent potentially interesting new drug targets, opening new avenues for the treatment and possibly also the prevention of auto-immune diabetes.

## Materials and Methods

### Antibodies and chemicals

Proinsulin was immunoprecipitated with guinea-pig anti insulin (Abcam). Westernblot analysis was performed with the following antibodies; H86 for proinsulin (Santa Cruz), anti Derlin-1 and Derlin-2 (MBL), anti VCP/p97 (BD transduction laboratories), anti-calreticulin (Thermo Scientific), 1E4 for GFP (Enzo Life Sciences), anti-actin clone c4 (Millipore), H86.4 anti transferrin receptor (Roche Diagnostics). Lactacystein was obtained from Enzo Life Sciences and ALLN (Calpain inhibitor I) from Sigma.

### Constructs

Preproinsulin cDNA was subcloned into the retroviral pLZRS-IRES-eGFP vector. A Derlin-1 expression construct was kindly provided by dr. Yihong Ye (Laboratory of Molecular Biology, NIDDK, NIH Bethesda, Maryland USA) and adjusted with side-directed mutagenisis to match Q9BUN8.1. A Derlin-2 plasmid was obtained from plasmID (#HsCD00330204). Both Derlins were subcloned behind the EF1α promoter in a bicistronic lentiviral expression vector containing a Zeocin resistance gene followed by T2A peptide and mAmetrine behind the hPGK promotor [13]. The shRNAs targeting Derlin 1 (TRCN0000062914), Derlin 2 [23 TRCN0000128182, #2 TRCN0000130112, #3 TRCN0000147427], p97 [23 TRCN0000004251, #2 TRCN0000004253] and HRD1 [23 TRCN0000034004, 2# TRCN0000034006] were obtained from Sigma and subcloned behind a U6 promotor into a lentiviral expression vector containing a puromycin resistance gene and mOrange.

### Cell lines

K562 stably expressing HLA-A*0201 were obtained from C Britten [23] and were maintained in IMDM (Lonza) and HEK293T cells in DMEM (Lonza). Cell culture medium was supplemented with 8% fetal calf serum (Sigma) containing 100 U/ml each of penicillin and streptomycin (Life Technologies) and 2 mM Glutamax I (Lonza) and kept in humidified incubators at 37°C containing 5% CO_2_.

### Western blot

Proinsulin molecules from cell lysates (20 mM MES, 50 mM Tris-HCl pH 7.4, 100 mM NaCl, 1% Triton X-100, 1 mM AESBF, 5 μg/ml leupeptin) were reduced with 50 mM DTT, treated with 100 mM N-Ethylmaleimode (NEM, Sigma) and separated using 12% NuPAGE Bis-Tris Gels (Invitrogen). After 70 minutes of transfer at 90 V, PVDF membranes (Milipore) were incubated for 60 minutes with 5% blotting milk in TBS (150 mM NaCl, 0.003 mM KCl, 25 mM Tris-HCl pH 7.5) and incubated 16 hours with the indicated antibody in 1% milk in TBS containing 0.05% Tween-20 (TBS-T). After extensive washing, membranes were incubated for 45 minutes with the secondary antibody in 1% milk in PBS-T and after incubation with ECL solutions (Pierce) membranes were exposed to film (GE healthcare).

### Viral transductions

Replication-deficient recombinant retroviruses were produced for preproinsulin expression using the Phoenix amphotropic packaging system, as described previously [47]. After 48–96 h, culture supernatants were harvested and filtered through a 0.45-μm-pore filter. K562 cells were infected with 1 ml retrovirus-containing medium in tissue culture dishes coated with 12 μg/ml RetroNectin. Transduction efficiency was examined by measuring eGFP expression and cells were sorted to obtain a 95–100% eGFP positive population using a FACS-Ariall sorter (Becton Dickinson). Derlin-1, Derlin-2, p97 and HRD1 overexpression and knockdown was realized by generating replication-deficient recombinant lentiviruses via linear PEI (1 mg/ml) cotransfection of HEK 293T cells with a pSico-based lentiviral vectors encoding the gene of interest or pKLO.1 based vector containing the shRNAs, as well as pCMV-VSVG, pMDLg-RRE, and pRSV-REV [48]. After 48–96 h, culture supernatants were harvested and filtered through a 0.45-μm-pore filter and preproinsulin expressing K562 were transduced with 100 μl lenti-virus containing medium during a 90 minutes spin infection at 33°C. Cells were selected with either 2 μg/ml puromycin (shRNA vectors) or 400 μg/ml Zeocin (Life Technologies) to obtain a pure population. Transduction efficiency was analyzed using a FACSCalibur flow cytometer (Becton Dickinson) using using FACS-DIVA (Becton Dickinson) and FlowJo (Tree Star, Ashland, OR) software FlowJo software (TreeStar).

### Pulse-chase analysis

The pulse-chase assay was performed as described before [49]. Briefly, K562-A2 cells were starved for 15–25 min in MEM without cysteine/methionine (ICN biomedicals) supplemented with Glutamax I (manufacturer) and were pulse-labeled for 15 min with ^35^S-methionine and cysteine (Easytag^TM^ Express Protein Labeling Mix, Perkin Elmer). Proinsulin was immunoprecipitated from the radiolabeled non-denaturing lysate (20 mM MES, 50 mM Tris-Cl pH 7.4, 100 mM NaCl, 1% Triton X-100, 1 mM AESBF, 5 μg/ml leupeptin) using guinea-pig anti-insulin antibody. The samples were reduced with 50 mM DTT, treated with 100 mM NEM and analyzed on 15% SDS-PA gel, prepared for fluorography, dried, and exposed to film (Kodak Biomax MS). Quantification was done with Quantity One software (Biorad)

### Peptide elution & mass spectrometry

Peptide elution was performed similar to van Lummel et al. [50]. Subsequently the HLA-peptides were analyzed via on-line C18-nano-HPLC-MS with a system consisting of an Easy nLC 1000 gradient HPLC system (Thermo, Bremen, Germany), and a Q-Exactive mass spectrometer (Thermo). Fractions were injected onto a homemade precolumn (100 μm × 15 mm; Reprosil-Pur C18-AQ 3 μm, Dr. Maisch, Ammerbuch, Germany) and eluted via a homemade analytical nano-HPLC column (15 cm × 50 μm; Reprosil-Pur C18-AQ 3 um). The gradient was run from 0% to 30% solvent B (10/90/0.1 water/ACN/FA v/v/v) in 120 min. The nano-HPLC column was drawn to a tip of ~5 μm and acted as the electrospray needle of the MS source. The Q-Exactive mass spectrometer was operated in top10-mode. Parameters were resolution 70,000 at an AGC target value of 3,000,000, maximum fill time of 100 ms (full scan), and resolution 35,000 at an AGC target value of 1,000,000/maximum fill time of 128 ms for MS/MS at an intensity threshold of 78,500. Apex trigger was set to 1 to 5 seconds, and allowed charges were 1–3. In a post-analysis process, raw data were converted to peak lists using Proteome Discoverer 1.4 (Thermo). For peptide identification, MS/MS spectra were submitted to the human IPI 3.87 database using Mascot Version 2.2.04 (Matrix Science) with the following settings: 10 ppm and 20 mmu deviation for precursor and fragment masses, respectively; no enzyme was specified. All reported hits were assessed manually, and peptides with MASCOT scores <35 were generally discarded.

## Supporting Information

S1 Fig MS2-spectra of the eluted peptides. Comparison of MS2-spectra of the eluted insulin peptide candidates and their synthetic counterparts for peptide HLVEALYLV, HLC(cys)GSHLVEA. In addition, the MS2-spectrum of peptide ALWGPDPAAA is shown. The precursor mass is indicated, and the best mascot ion score. Fragments are annotated. I_L_ = immonium ion of L, I_Y_ = immonium ion of Y, etc. The charge of the ions is indicated if >1. * = ammonia loss.
(TIF)
