# Supplementary figures and images for: Proteasomal Degradation of Proinsulin Requires Derlin-2, HRD1 and p97
Source: PLoS One. 2015 Jun 24;10(6):e0128206. doi: 10.1371/journal.pone.0128206 (PMC4479611; doi:10.1371/journal.pone.0128206)

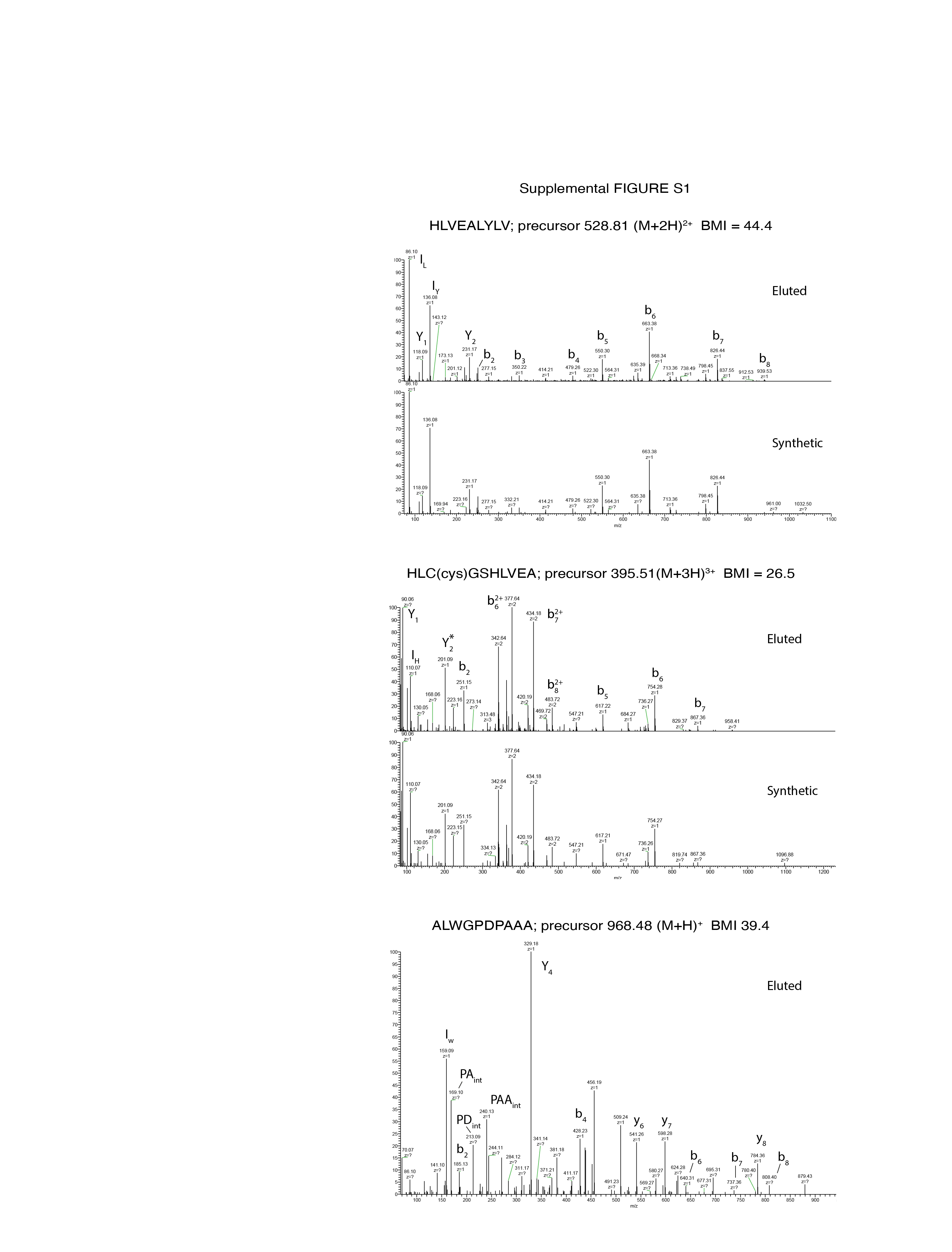

Supplement: S1 Fig — Comparison of MS2-spectra of the eluted insulin peptide candidates and their synthetic counterparts for peptide HLVEALYLV, HLC(cys)GSHLVEA. In addition, the MS2-spectrum of peptide ALWGPDPAAA is shown. The precursor mass is indicated, and the best mascot ion score. Fragments are annotated. IL = immonium ion of L, IY = immonium ion of Y, etc. The charge of the ions is indicated if >1. * = ammonia loss. (TIF) [file pone.0128206.s001.tif]
